# CDC20 inhibitor Apcin inhibits embryo implantation in vivo and in vitro

**DOI:** 10.1002/cbf.3550

**Published:** 2020-05-26

**Authors:** Chuanjia Guo, Fandou Kong, Yunyi Lv, Na Gao, Xiaoxin Xiu, Xiaojing Sun

**Affiliations:** ^1^ Department of Gynecology and Obstetrics First Affiliated Hospital of Dalian Medical University Dalian China

**Keywords:** adhesion, Apcin, CDC20, implantation, proliferation

## Abstract

For successful implantation, endometrial receptivity must be established. The high expression of CDC20 in many kinds of malignant tumours has been reported, and it is related to the occurrence and development of tumours. According to these functions, we think that CDC20 may also play important roles in the process of embryo implantation. To prove our hypothesis, we observed the distribution and expression of CDC20 in mouse and human early pregnancy. The effect of E2 and/or P4 on the expression of CDC20 in human endometrial cells was detected by Western blot. To further explore whether CDC20 is an important factor in adhesion and proliferation. The results showed that the expression of CDC20 in the uterus and menstrual cycle of early pregnant mice was spatiotemporal. E2 can promote the expression of CDC20. On the contrary, P4 and E2 + P4 inhibited the expression of CDC20. We also detected the proliferation and adhesion of human endometrial cells. We found that the inhibition of CDC20 with its inhibitor Apcin could reduce the adhesion rate and proliferation ability to RL95‐2 and HEC‐1A cells, respectively. Inhibiting CDC20 by Apcin could interfere the embryo implantation of mouse. It is suggested that CDC20 may play an important role in the process of embryo implantation.

**Significance of the study:**

Embryo implantation is an extremely complex and delicate process, including identification, localisation, adhesion and invasion between embryo and endometrium. Studies have shown the process of embryo implantation is very similar to that of tumour invasion. CDC20 is a cancer‐promoting factor. We found CDC20 is spatially and spatially expressed in mouse and human menstrual cycles and is regulated by oestrogen and progesterone. Apcin can inhibit the adhesion of JAR cells and embryo implantation of mouse. CDC20 may provide a new way to improve the success rate of assisted reproduction.

## 1 INTRODUCTION

Embryo implantation is a complex and delicate process, which requires the coordination of embryo and endometrium in time and space. In mammals, the formation of the “implantation window period” is a key step in successful implantation.^1^ Researchers have identified a variety of biomolecules involved in the formation of the “implantation window,” including cytokines, growth factors, adhesion molecules and extracellular matrix complexes.^2^, ^3^, ^4^ They play an important role in the proliferation and differentiation of endometrium into a state that is easy to receive blastocyst recognition and adhesion.^5^, ^6^

Estrogen (E2) and progesterone (P4) are the most important factors, which are in the upstream of many factors.^7^ Human endometrium has been regulated by E2 and P4 in different menstrual cycles, which are the main regulators of periodic repair, proliferation and exfoliation of endometrium.^8^, ^9^ E2 produced by ovary is mainly expressed in proliferative endometrium, which promotes endometrial proliferation and follicular maturation, while P4 produced by corpus luteum antagonises estrogen‐mediated proliferation and growth. The menstrual cycle is divided into follicular phase (proliferative phase), luteal phase (secretory phase) and menstrual phase. During the secretory phase, the expression of progesterone increases, and the glandular epithelium changes from relatively inactivated cells full of free ribosomes to activated polar cells, such as giant mitochondria, the enrichment of intracellular glycogen and glycoproteins and complex nuclear channels. Morphologically, during the secretory phase, the concentration of progesterone increased, the glandular epithelium became distorted, and the luminal epithelium became huge; stromal cells became edema, enlarged and polygonal due to the increase of capillary permeability. The process of this transformation is decidualisation, which occurs in the mid‐secretory phase, the upper 2/3 of the endometrium. If embryo implantation occurs, the above response will be enhanced. Decidual cells express specific proteins such as insulin‐like growth factor‐binding protein‐1 (IGFBP‐1), insulin‐like binding factor growth protein‐1 (IGF‐1) and prolactin (PRL), which promote the transition of endometrium to receptive state and prepare for embryo implantation.^10^ If you are not pregnant, the levels of E2 and P4 will decrease due to the recovery of lutein, and the upper 2/3 of the endometrium will fall off, that is, menstruation. The implantation window phase is mediated by P4, while E2 locally regulates the production of cytokines, growth factors, HOX transcription factors and autocrine or paracrine prostaglandins (PG).

Ubiquitin‐proteasome system (UPS) plays an important role in a variety of biological processes, including cell cycle, proliferation, apoptosis, implantation^11^ and survival‐related processes. As an ubiquitin ligase, anaphase‐promoting complex/cyclone (APC/C) plays a role in the cell cycle. RING finger E3 ubiquitin ligase cell division cycle 20 homologue (CDC20) serves as an activator of APC/C during the transition from metaphase to anaphase of cell division.^12^ CDC20 has been proved to be highly expressed in a variety of malignant tumours and is related to tumorigenesis and progression.^13^ A small molecular Apcin (APC inhibitor) can bind CDC20 and block substrate recognition, resulting in competitive inhibition of ubiquitination of CDC20 substrates.^14^

In recent years, endometrial cancer cells and human choriocarcinoma cells are often used to represent endometrial embryos in vitro models of embryo implantation. RL95‐2 is a human endometrial cancer cell, which is regarded as endometrial high receptive cell because of its high adhesion rate to human choriocarcinoma cell JAR, while HEC‐1A cell is a human endometrial adenocarcinoma cell with low adhesion rate to human choriocarcinoma cell JAR, and is regarded as endometrial low receptive cell because of its high adhesion rate to human choriocarcinoma cell line JAR.^15^, ^16^

In this study, we observed the expression of CDC20 in human and mouse menstrual cycle and preimplantation uterus. We also found the effects of ovarian steroids E2 and P4 on the expression of CDC20 in RL95 and HEC‐1A cells. We studied the effect of CDC20 inhibitor Apcin on JAR adhesion.

## 2 MATERIALS AND METHODS

### 2.1 Tissue collection

The agreement on human research is approved by the Ethics Committee of Dalian Medical University. All women who participated in the study gave written consent. With the consent of the patient, the endometrial samples of women who had given birth normally and had regular menstrual cycle and underwent hysterectomy were obtained.

### 2.2 Animals experiments

The adult C57BL/6 female mice were 8 weeks old, weighing 20 to 25 g, and the male was 12‐week‐old, weighing 40 to 50 g. All experimental procedures involve in the mouse studies were approved by the Institutional Review Board in Dalian Medical University. The mice live in an environment where the temperature is 22°C, the humidity is 60%, the light is controlled (12 hours of light, 12 hours of darkness) and water and food are available at will. Female rats are placed together with male rats (one female and one male in each cage). The next morning to check whether the woman has a vaginal plug, if the vaginal plug comes out, it is defined as D1. The mice were sacrificed at D2 to D5.

To investigate the role of CDC20 in embryo implantation, pregnant mice were randomly divided into two groups. On the second day of pregnancy (D2), Apcin was injected into the right uterine horn and the vechile into the left uterine horn. All mice were euthanised on the D9 of pregnancy, and the number of transferred embryos was counted.

### 2.3 Cell experiments

JAR cells were cultured in DMEM/F12 medium containing 10% fetal bovine serum and double antibodies (penicillin 100 U amp ml, streptomycin 100 μg/mL). RL95‐2 cells were cultured in DMEM/F12 medium containing 10% fetal bovine serum, double antibodies (penicillin 100 U/mL, streptomycin 100 μg/mL) and insulin (5 μg/mL). HEC‐1A cells were cultured in MCCOY'S 5A medium with 10% fetal bovine serum and double antibodies. The cells were cultured at 37°C, 5% CO_2_ and 90% humidity. The cells change the fluid every 2 or 3 days according to the cell density. When the cell grows to 80%, it is digested and passaged with 0.25% trypsin according to the experiment. When the RL95‐2 and HEC‐1A cells grow to 80%, different concentrations of E2 (0, 0.1, 1, 10 μmol/L) and P4 (0, 1, 10, 100 μmol/L) are added and cultured for 48 hours. Then the cells were treated with E2 (10 μmol/L) and P4 (100 μmol/L) for different time (0, 24, 48, 72 hours). The protein extracted from the cells was collected and the expression of CDC20 was detected.

### 2.4 Western blot and Immunohistochemistry

Follow the steps to complete the experiment of Western blot and Immunohistochemistry.

### 2.5 Cell adhesion assay

The endometrial cells RL95‐2 and HEC‐1A were placed in the culture well of a 96‐well plate, and the experiment was carried out after the cells grew to 90% and fused to form a monolayer cell. JAR cells were pretreated with CellTracker fluorescent Green CMFDA for 1 hour. The treated JAR cells were digested with 0.25% trypsin, and JAR cells were collected and counted. The endometrial cells RL95‐2 and HEC‐1A culture medium were discarded and added to the culture medium of JAR cells. The culture conditions were the same as JAR cells, and co‐cultured at 37°C with 5% CO_2_, 90% humidity for 1 hour. The OD values of each group were detected by fluorescence enzyme labelling instrument, and parallel wells were set in each group. The excitation wavelength is 492 nm, and the emission wavelength is 518 nm. Adhesion rate of JAR cells = (fluorescence OD value of adhered JAR cells/fluorescence OD value of total JAR cells added) × 100%.

### 2.6 Statistical analysis

The analysis was performed using GraphPad Prism 8.0.1 statistical software, and each experiment was repeated three times. The results were expressed as mean ± SD, and one‐way analysis of variance was used between the comparison groups. The difference of */#*P* < .05, **/##*P* < .05, ***/###*P* < .001 were considered as a significant difference.

## 3 RESULTS

### 3.1 Expression of CDC20 in human endometrial tissues

**FIGURE 1:**
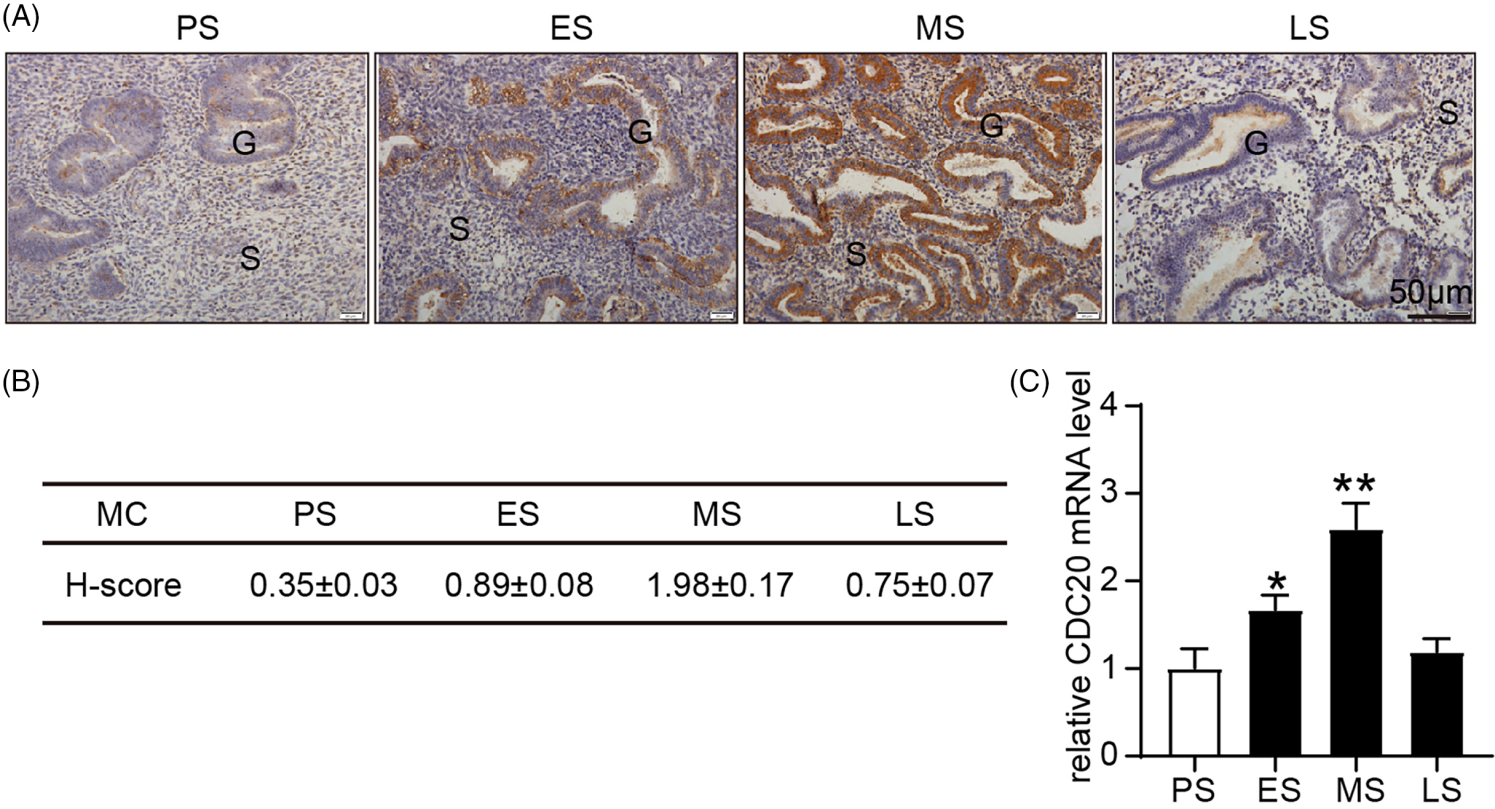
Expression of CDC20 in human endometrial tissues. A, The expression of CDC20 in the menstrual cycles in human endometrial tissues; B, The image analysis of CDC20 in the menstrual cycles in human endometrial tissues; C, The mRNA expression of CDC20 in in the menstrual cycles in human endometrial tissues. *P < .05, **P < .01. stromal cells (S), glandular epithelium (G), menstrual cycle (MC), proliferative phase (PS), early secretory phase (ES), mid‐secretory phase (MS), late‐secretory phase (LS)

In order to detect the changes of CDC20 in the menstrual cycle, we detected the expression of CDC20 in human endometrium by immunohistochemical assay. The results showed that CDC20 was not expressed in the proliferative phase, but began to express in the secretory phase, reached the highest expression in the mid‐secretory phase, and was mainly expressed in the gland rather than in the matrix (Figure 1A‐C).

### 3.2 Expression of CDC20 in mouse endometrial tissues

**FIGURE 2:**
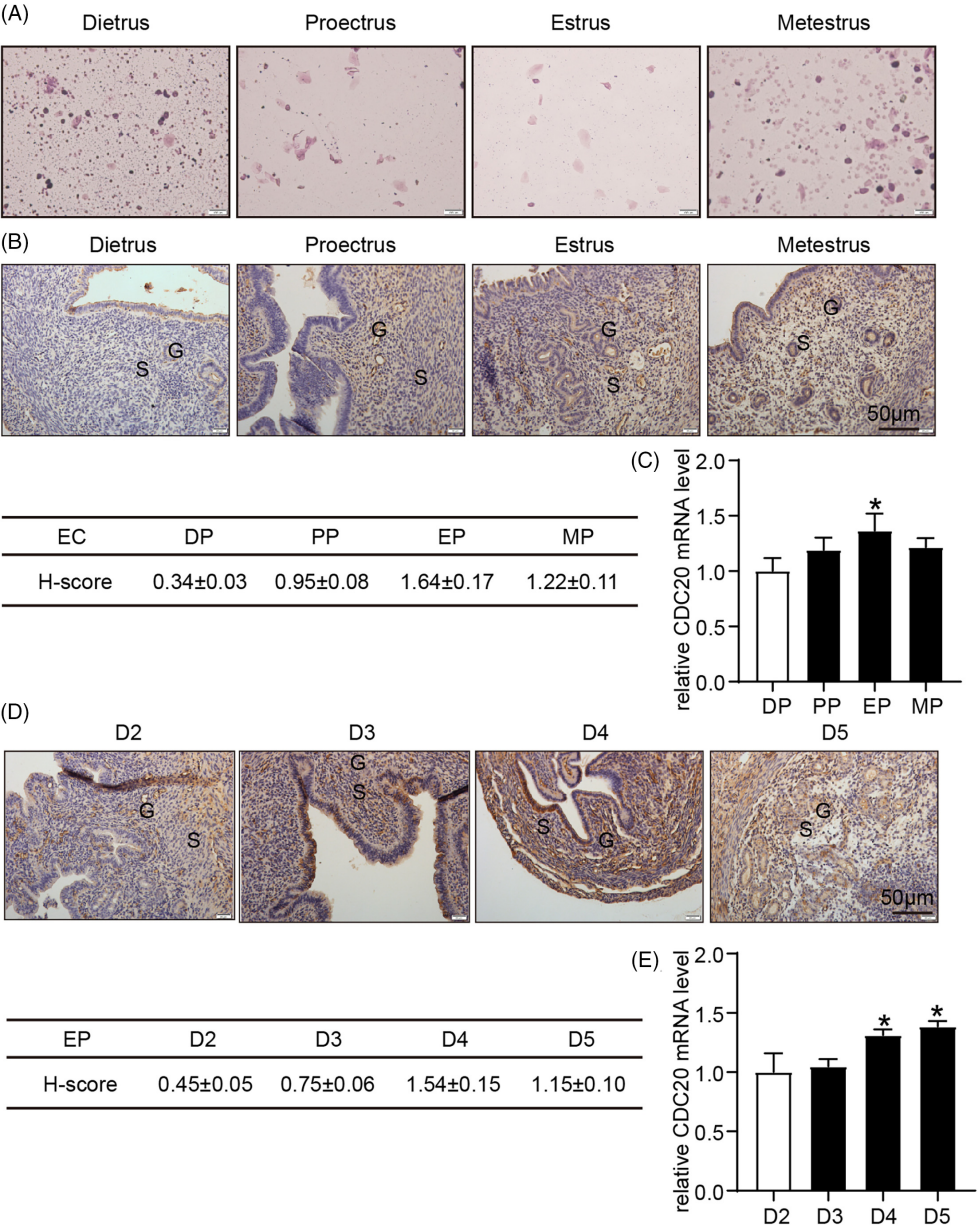
Expression of CDC20 in mouse endometrial tissues. A, Vaginal cytology representing each stage of estrous by HE staining; B, The expression and image analysis of CDC20 during estrous cycle in mouse uterus; C, The mRNA expression of CDC20 during estrous cycle in mouse uterus; D, The expression and image analysis of CDC20 during early pregnancy in mouse uterus; E, The mRNA expression of CDC20 during early pregnancy in mouse uterus. *P < .05. diestrus period (DP), proestrus period (PP), estrus period (EP), metestrus period (MP), estrous cycle (EC) and early pregnant (EP)

The estrus cycle of mice is generally 4 to 5 days. We use vaginal smears to select four periods: diestrus, proestrus, estrus and metestrus. During diestrus, vaginal smears are mostly white blood cells and a large amount of mucus, which is due to the thinning of vaginal mucosa and the dissociation of white blood cells from the mucosa, so that vaginal smears are all white blood cells. In proestrus, most of the vaginal smears were nucleated epithelial cells, some were single or flaky, and there were a small number of white blood cells. In proestrus, most of the vaginal smears were nucleated keratinised squamous cells with a small amount of leukocytes and epithelial cells. In the later stage of estrus, the vaginal smears contained keratinised squamous cells and leukocytes, and a small amount of nucleated epithelial cells (Figure 2A).

Next, we examined the expression of CDC20 in mice during the estrous cycle. The results showed that CDC20 began to express in proestrus, reached its peak in estrus and decreased in metestrus. Different from human tissues, CDC20 was expressed in mouse endometrial glands and stroma (Figure 2B). The realtime PCR showed that the mRNA expression of CDC20 reached its peak in estrus (Figure 2C). Finally, we used immunohistochemistry to detect the expression of CDC20 in naturally conceived mice. With the increase of pregnancy days, the expression of CDC20 also increased. CDC20 was expressed in glands and stroma at D4. On D5, the expression of CDC20 was mainly located in the stroma (Figure 2D). The realtime PCR showed that the mRNA expression of CDC20 was gradually increased (Figure 2E).

### 3.3 Effect of E2 and P4 on the expression of CDC20 in HEC‐1A and RL95‐2 cells

**FIGURE 3:**
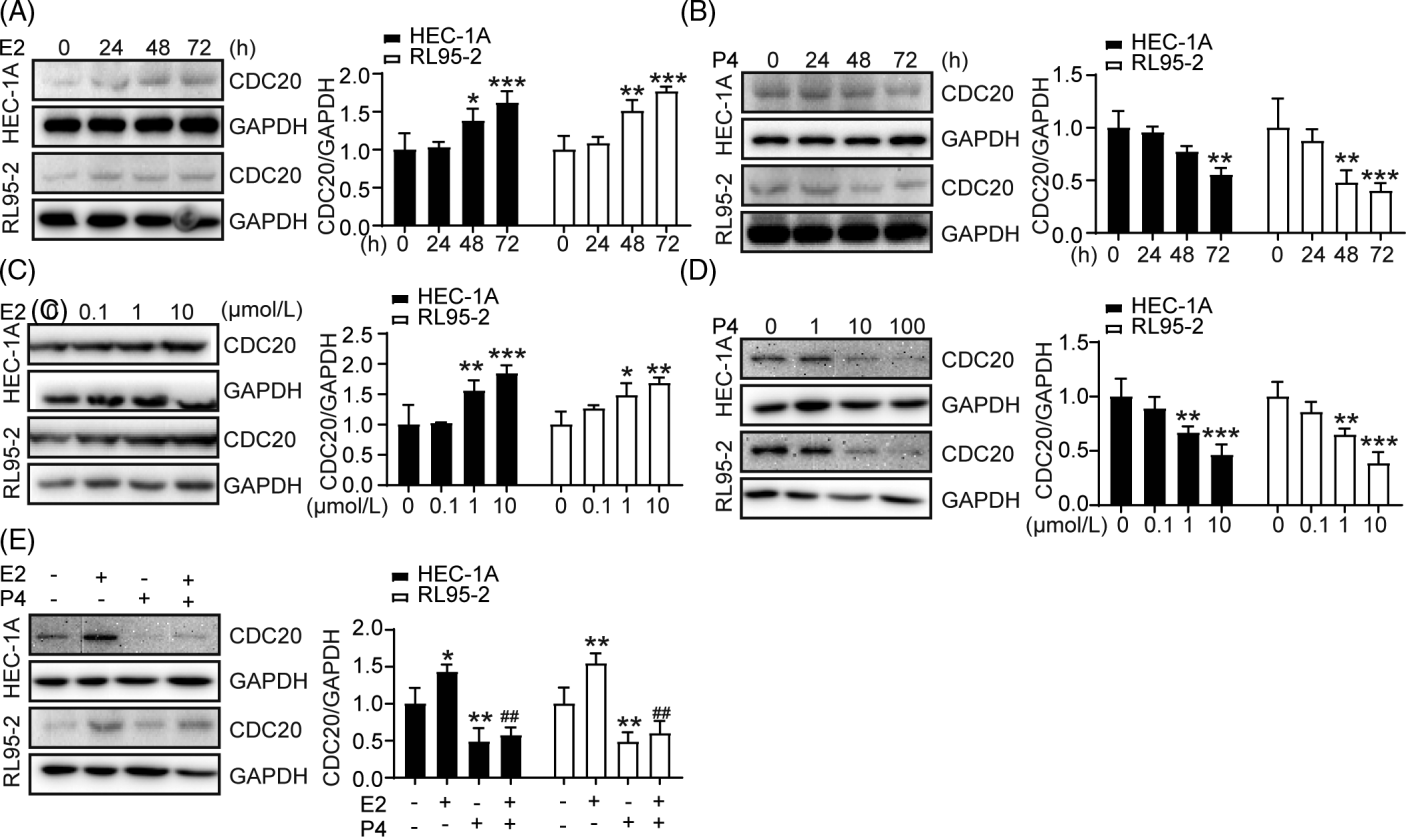
Effect of E2 and P4 on the expression of CDC20 in HEC‐1A and RL95‐2 cells. A, The expression of CDC20 in HEC‐1A and RL95‐2 cells treated by E2 with different concentration; B, The expression of CDC20 in HEC‐1A and RL95‐2 cells treated by P4 with different concentration; C, The expression of CDC20 in HEC‐1A and RL95‐2 cells treated by E2 with different time; D, The expression of CDC20 in HEC‐1A and RL95‐2 cells treated by P4 with different time; E, The expression of CDC20 treated by E2 and/or P4. *P < .05, **P < .01 vs control; ##*P* < .01 vs E2

In this experiment, high and low receptive cells of human endometrium were stimulated with different concentrations of E2 for 48 hours, the results showed that the expression of CDC20 increased with the increase of E2 concentration (Figure 3A), and then we stimulated the two types of cells with different time in 10 μM of E2, the results showed that the expression of CDC20 was highest in the two types of cells (Figure 3C). We stimulated the two types of cells with different concentrations of P4 for 48 hours, the results showed that the expression of CDC20 was significantly down‐regulated with the increase of P4 concentration (Figure 3B), and then treated with 100 μM P4 with different time, the results showed that the longer the action time, the lower the expression of CDC20 (Figure 3D). We also treated the two types of cells with 10 μM E2 and 100 μM P4, and the results showed that the expression of CDC20 in the combination of E2 and P4 was lower than that in the P4 group (Figure 3E).

### 3.4 CDC20 inhibitor Apcin hindered the proliferative and receptive potentials in HEC‐1A and RL95‐2 cells and the embryo implantation

**FIGURE 4:**
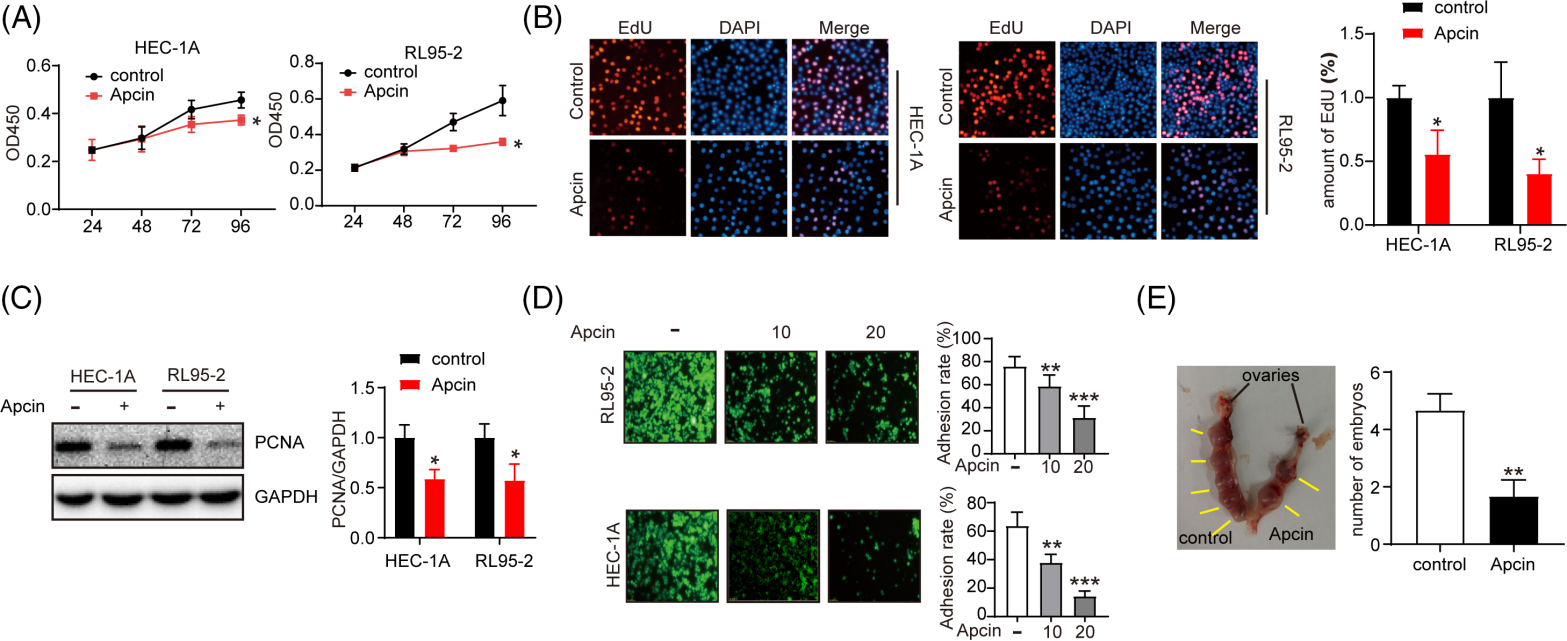
CDC20 inhibitor Apcin hindered the ability of proliferation and reception potentials in HEC‐1A and RL95‐2 cells and the embryo implantation. A, The CCK‐8 assay results showed the effects on Apcin in HEC‐1A and RL95‐2 cells in cell proliferation; B, Representative images of DAPI (blue), EdU (red) and Merge (pink) showed the effects on Apcin in HEC‐1A and RL95‐2 cells in cell proliferation; C, The protein expression levels of PCNA showed the effects on Apcin in HEC‐1A and RL95‐2 cells in cell adhesion; D, Adhered JAR cells (Green) to HEC‐1A and RL95‐2 cells with different concentration of Apcin; E, The number of implanted embryos were significantly inhibited in the group injected with Apcin compared with control group. *P < .05, **P < .01, ***P < .001

The proliferation of endometrial cells is a prerequisite for the establishment of endometrial receptivity. Therefore, we examined the regulatory effect of Apcin on the proliferation of endometrial cells. Apcin and control were added to RL95‐2 and HEC‐1A cells. CCK‐8 and EDU experiments showed that Apcin could inhibit the proliferation of HEC‐1A and RL95‐2 cells (Figure 4A,B). At the same time, the expression level of cell proliferation protein was detected by Western blot. The results showed that after the addition of Apcin, the expression of PCNA decreased (Figure 4C). The role of CDC20 in cell adhesion was analysed by using an in vitro implantation model composed of trophoblast JAR, uterine epithelial RL95‐2 cells or HEC‐1A cells. The adherent trophoblast cells were observed by fluorescence staining, and the attachment rate was analysed (Figure. 4D). The results showed that Apcin significantly decreased the adhesion rate of trophoblast cells to RL95‐2 cells and HEC‐1A cells. Then, in order to determine whether CDC20 plays a role in embryo implantation, we injected Apcin into mouse uterus in D2. As shown in Figure 4E, the number of embryos in the Apcin group were significantly lower than that in the control group (Figure 4E).

## 4 DISCUSSION

In the complex process of embryo implantation, it can be divided into localisation, adhesion implantation and trophoblast invasion.^17^ Implantation failure is not only an important cause of female infertility, but also one of the obstacles to the development of assisted reproductive technology. Embryo implantation can only occur in the “implantation window stage.”^18^, ^19^ Through the mutual regulation of adhesion molecules, immune cells, cytokines, growth factors and chemokines, blastocysts are recognised and received by the maternal uterus.

Immunohistochemical method was used to detect the expression of CDC20 in human endometrium during the menstrual cycle. It was found that the expression of CDC20 increased gradually in the proliferative phase of endometrium, reached a peak in the middle stage of secretion and decreased in the late stage of endometrial secretion. This phenomenon indicates that CDC20 plays a role in endometrial changes with the menstrual cycle. In the immunohistochemical experiment of D1 to D5 endometrium of pregnant mice and estrus cycle of mice, it showed that the endometrium of pregnant mice was highly expressed during per‐implantation and highest expression in Estrus. Therefore, we speculate that CDC20 plays an important role in promoting endometrial proliferation and regulating endometrium entering the implantation window during the pre‐implantation period. Furthermore, we added Apcin in HEC‐1A and RL95‐2 cells to detect the effect of CDC20 on cell proliferation and adhesion. The results showed that Apcin could inhibit the proliferative and receptive potentials in HEC‐1A and RL95‐2 cells.

The endometrium becomes receptive to the implantation of trophoblast cells and is regulated by ovarian sex hormones, estrogen and progesterone.^8^ In humans, the up‐regulation of estrogen expression occurs in the follicular phase (proliferative phase), which can promote the proliferation and growth of epithelial and stromal cells; differentiated granulosa cells in the ovary enter the corpus luteum, resulting in an up‐regulated expression of progesterone. In turn, the menstrual cycle enters the luteal phase (secretory phase). During this period, the endometrium stopped proliferation and mainly differentiated into decidual tissue under the action of progesterone. The human menstrual cycle is about 28 to 30 days, while the mouse menstrual cycle is relatively short, only 4 to 5 days, which can be divided into proestrus, estrus, diestrus and interestrus.^20^

Hormones mainly function by binding to their receptors, and their receptors are divided into membrane receptors and nuclear receptors, of which the latter plays a major role. The nuclear receptors of estrogen include estrogen receptor α (ERα) and estrogen receptor β (ERβ). These two receptors perform two completely opposite functions. ERα was mainly expressed in breast glands, pituitary, hypothalamus, ovarian parietal cells and uterus, while ERβ was mainly expressed in ovarian granulosa cells, lungs and prostate. ERα was highly expressed in the glandular epithelium and stromal cells in the proliferative phase, but low in the secretory phase. ERα knockout mice had uterine hypoplasia and could not conceive, while ERβ knockout mice could conceive normally. Progesterone also has two nuclear receptors‐PR‐A (progesterone receptor‐A, progesterone receptor‐A) and PR‐B (progesterone receptor‐B, progesterone receptor‐B) and have different functions. PR‐A inhibited endometrial proliferation, and PR‐A knockout mice showed endometrial proliferation, implantation failure and decidual repair. However, the development of mammary glands was blocked only in the mice after PR‐B knockout.

In in vitro experiments we added different concentrations of E2 and P4 to treat RL95‐2 and HEC‐1A cells, and the results showed that E2 could up‐regulate the expression of CDC20 in the two types of cells, while P4 and the combination of E2 and P4 down‐regulated the expression of CDC20.

To sum up, we revealed the expression of CDC20 in human and mouse menstrual cycle and preimplantation uterus. The high expression of CDC20 in human and mouse endometrium suggests that CDC20 may be involved in the development of early pregnancy, especially in endometrial proliferation. As a new implantation‐related molecule, the clear cutting mechanism of CDC20 needs to be further studied. CDC20 may provide a new way to improve the success rate of assisted reproduction.

## CONFLICT OF INTEREST

The authors declare no conflicts of interest.

## Data Availability

All data generated or analysed during this study are included in this published article.
